# A Comparative Mixed Longitudinal Evaluation of Doxycycline Solution as an Aligner Rinse Compared With Conventional Mouthwashes on Microbial, Esthetic, and Inflammatory Outcomes During Orthodontic Tooth Movement

**DOI:** 10.7759/cureus.92767

**Published:** 2025-09-20

**Authors:** Siddharth S Sonwane, Shweta S Sonwane (Kamble), Purvi Awasthi

**Affiliations:** 1 Orthodontics, Mansarovar Dental College Bhopal, Bhopal, IND; 2 Oral and Maxillofacial Surgery, Government Dental College Nagpur, Nagpur, IND; 3 Orthodontics and Dentofacial Orthopedics, Mansarovar Dental College Bhopal, Bhopal, IND

**Keywords:** aligners, antimicrobial orthodontic therapy, colony-forming units (cfus), doxycycline, gingival crevicular fluid biomarkers, gingival crevicular fluid (gcf), orthodontic tooth movement

## Abstract

Introduction: Clear aligners are vulnerable to microbial colonization, discoloration, and inflammation-associated delays in orthodontic tooth movement. This study explores the use of sub-antimicrobial-dose doxycycline as a novel aligner disinfectant, owing to its combined antimicrobial, anti-inflammatory, and osteogenic effects.

Objective: This study aims to compare the clinical, microbiological, and biological efficacy of 0.1% doxycycline solution versus 0.2% chlorhexidine gluconate in aligner therapy.

Materials and methods: A mixed longitudinal cohort design was employed, enrolling 60 patients undergoing aligner therapy. Participants received alternating aligners soaked in doxycycline (group A) and chlorhexidine (group B). Clinical indices, colony-forming units (CFUs), gingival crevicular fluid (GCF) alkaline phosphatase (ALP), and aligner transparency were evaluated at baseline, two weeks, one month, three months, and six months. Random allocation was performed using a computer-generated sequence. Outcome assessors were blinded. Microbial cultures, ALP assays, and spectrophotometric evaluations were conducted using standardized protocols.

Results: Group A exhibited significantly lower plaque and gingival indices, reduced CFUs, enhanced aligner clarity, and elevated ALP levels compared to group B (p < 0.01). Pearson’s correlation showed a strong inverse relationship between ALP and both CFUs and the gingival index.

Conclusions: Doxycycline demonstrated superior microbial control, esthetic maintenance, and biological stimulation, supporting its potential role as a dual-action adjunct in aligner-based orthodontics. Further multicenter studies are recommended to validate these findings.

## Introduction

Clear aligner therapy is increasingly favored in orthodontics for its aesthetic benefits, user convenience, and facilitation of oral hygiene [1]. However, the continuous intraoral wear of aligners, typically exceeding 22 hours daily, creates an environment favorable to microbial colonization, plaque accumulation, and gingival inflammation [2]. Moreover, frequent exposure to pigmented foods and inadequate hygiene practices can diminish the transparency of aligners, potentially affecting patient satisfaction and treatment compliance [1-3].

Chlorhexidine gluconate (CHX), a widely used antimicrobial mouthwash in orthodontics, has demonstrated effective microbial control [4]. Nonetheless, its long-term use is associated with adverse effects such as altered taste sensation, oral mucosal irritation, and staining of both dentition and aligners [5]. These limitations necessitate the exploration of alternative agents that offer comparable antimicrobial efficacy without compromising aesthetics [1,3,5].

Doxycycline, a semi-synthetic tetracycline derivative, has shown promise as a sub-antimicrobial dose therapy (SADD) with both anti-inflammatory and osteogenic properties. At low concentrations, doxycycline inhibits matrix metalloproteinases (MMPs), thereby reducing collagen degradation and supporting periodontal tissue integrity [5]. It also contributes to bone remodeling through the upregulation of biomarkers, such as alkaline phosphatase (ALP), which may enhance orthodontic tooth movement [5]. Recent studies further suggest that doxycycline may prevent biofilm formation on oral appliances while preserving aligner clarity due to its low staining potential [6].

Despite encouraging evidence in periodontal contexts, the role of doxycycline in aligner-based orthodontic treatment remains underexplored. Addressing this gap, the present study evaluates doxycycline as a novel daily aligner rinse, comparing its clinical, microbial, and biological efficacy with that of CHX [7-8].

The objective of this mixed longitudinal study is to evaluate plaque index (PI) and gingival index (GI), microbial load, aligner esthetics, and gingival crevicular fluid (GCF) ALP levels in patients using either doxycycline or CHX as an aligner disinfectant. By examining both esthetic and biological parameters, this study aims to inform clinical strategies for optimizing aligner hygiene while enhancing treatment efficiency.

## Materials and methods

Study design and ethical approval

This prospective, intra-subject, mixed-method longitudinal study was conducted over six months in the Department of Orthodontics at a tertiary dental care center. Ethical clearance was obtained from the Institutional Ethics Committee of Mansarovar Dental College, Hospital, and Research Centre (approval number: MDCH/IEC34/2025/ortho/Micro-62, dated 18 December 2024), and all procedures adhered to the Declaration of Helsinki. Written informed consent was collected from each participant prior to enrollment.

Sample size and sampling technique

A total of 40 participants aged 15 to 30 years undergoing non-extraction aligner therapy for mild-to-moderate malocclusion were included. Sample size was calculated using G*Power software (Heinrich-Heine-Universität Düsseldorf, Germany), targeting 80% power and α = 0.05, referencing prior studies evaluating microbial and biochemical variables. Participants were selected using simple random sampling through a computer-generated randomization sequence.

Inclusion and exclusion criteria

Participants were eligible if they were between 15 and 30 years of age, in good systemic and periodontal health, and prescribed full-arch aligner therapy. Those with recent antibiotic or anti-inflammatory use (within 30 days), smokers, pregnant or lactating individuals, or those with active periodontal disease were excluded.

Study protocol

Each participant received two identical sets of thermoplastic aligners. One set was used with a 0.1% doxycycline hydrochloride solution, freshly prepared each day in distilled water and stored in an amber-colored glass container. The other set was disinfected using 0.2% CHX mouthwash. Aligners were worn in alternating two-week cycles to minimize carryover effects. Participants were instructed to wear aligners for at least 22 hours per day while maintaining consistent oral hygiene. The disinfection sequence (doxycycline or CHX first) was randomized.

Blinding and calibration

Outcome assessors were blinded to group allocation. All examiners underwent calibration sessions using standardized indices to ensure consistency and inter-examiner reliability.

Clinical and esthetic assessments

Plaque index (PI) and gingival index (GI) were recorded using the Silness and Löe criteria, respectively. Aligner clarity was evaluated using a Visual Analog Scale and spectrophotometric analysis based on the Lab* color space.

Microbial evaluation

Aligner surfaces were swabbed and cultured on blood agar and Mitis Salivarius agar to quantify colony-forming units (CFUs) of *Streptococcus mutans*, *Lactobacillus species*, and *Candida albicans*. Selected pooled samples also underwent 16S rRNA gene sequencing to assess microbial diversity.

Biochemical analysis

GCF samples were collected from mesiobuccal and distobuccal sites of the upper first premolars using microcapillary pipettes. ALP activity was determined using a colorimetric assay with a semi-automated analyzer.

Tooth movement tracking

Digital intraoral scans were obtained at baseline and monthly intervals. Superimpositions and three-dimensional tooth movement analyses were performed using OrthoAnalyzer software (3Shape A/S, Copenhagen, Denmark) to assess the rate and direction of tooth movement.

Statistical analysis

Data were analyzed using SPSS Statistics version 25.0 (IBM Corp. Released 2017. IBM SPSS Statistics for Windows, Version 25.0. Armonk, NY: IBM Corp.). Repeated measures ANOVA and paired t-tests were used for intra- and inter-group comparisons. Microbial diversity indices were computed using QIIME2. Pearson’s correlation coefficient was applied to explore relationships among clinical, microbiological, and biochemical variables. A p-value of <0.05 was considered statistically significant.

## Results

All 40 participants completed the six-month study period without any adverse events or dropouts. The alternating use of aligners allowed for effective intra-subject comparison between the doxycycline and chlorhexidine interventions.

Plaque and gingival indices

At 12 weeks, participants using doxycycline-soaked aligners showed significantly lower PI and GI scores compared to those using chlorhexidine (p < 0.01). As shown in Table 1, the mean PI in the doxycycline group was 0.70 ± 0.16, and GI was 0.50 ± 0.14, whereas the chlorhexidine group showed higher values (PI: 1.20 ± 0.22; GI: 1.00 ± 0.19). The control group had the highest scores (PI: 1.90 ± 0.28; GI: 1.70 ± 0.25).

**Table 1:** PI and GI at 12 weeks across groups PI: plaque index, GI: gingival index, SD: standard deviation

Group	PI (mean ± SD)	GI (mean ± SD)
Control (A)	1.90 ± 0.28	1.70 ± 0.25
Chlorhexidine (B)	1.20 ± 0.22	1.00 ± 0.19
Doxycycline (C)	0.70 ± 0.16	0.50 ± 0.14
p-value	<0.01	<0.01

Microbial load on aligner surfaces

Bacterial CFUs (×10³) on aligner surfaces were lowest in the doxycycline group (30 ± 5), followed by chlorhexidine (75 ± 8), and highest in the control group (120 ± 10), as detailed in Table 2. Microbiological cultures revealed that aligners treated with doxycycline had significantly reduced colonization by *Streptococcus mutans*, *Lactobacillus* spp., and *Candida albicans* (p < 0.01).

**Table 2:** CFUs recovered from aligner surfaces at 12 weeks CFUs: colony-forming units

Group	Mean CFUs (×10³)	Predominant microorganisms detected
Control (A)	120	*Streptococcus mutans*, *Lactobacillus* spp., *Candida albicans*
Chlorhexidine (B)	75	*Streptococcus* spp., *Lactobacillus* spp.
Doxycycline (C)	30	*Streptococcus* spp. (minimal)
p-value	<0.01	-

Aligner transparency and staining

Aligner esthetics were better preserved in the doxycycline group, where only 10% of aligners showed moderate to severe discoloration, in contrast to 40% in the chlorhexidine group and 70% in the control group. This data is presented in Table 3. Spectrophotometric analysis confirmed significantly less color change in the doxycycline group (p < 0.01).

**Table 3:** Percentage of aligner staining and transparency loss by group

Group	Moderate to severe staining (%)	Mild or no staining (%)
Control (A)	70	30
Chlorhexidine (B)	40	60
Doxycycline (C)	10	90
p-value	<0.01	-

GCF ALP levels

GCF analysis demonstrated that ALP levels progressively increased in the doxycycline group across time points. As presented in Table 4, by 12 weeks, ALP levels reached 32.4 ± 2.8 IU/L in the doxycycline group, compared to 26.7 ± 2.5 IU/L in the chlorhexidine group and 21.5 ± 3.1 IU/L in controls (p < 0.001). This indicates a stimulatory effect on osteoblastic activity.

**Table 4:** GCF ALP levels over time GCF: gingival crevicular fluid, ALP: alkaline phosphatase

Time point	Control (A)	Chlorhexidine (B)	Doxycycline (C)	p-value
Week 0	20.0 ± 1.5	20.1 ± 1.4	20.2 ± 1.3	>0.05
Week 6	20.8 ± 1.7	23.5 ± 2.0	27.3 ± 2.2	<0.01
Week 12	21.5 ± 3.1	26.7 ± 2.5	32.4 ± 2.8	<0.001

Correlation analysis

**Figure 1:**
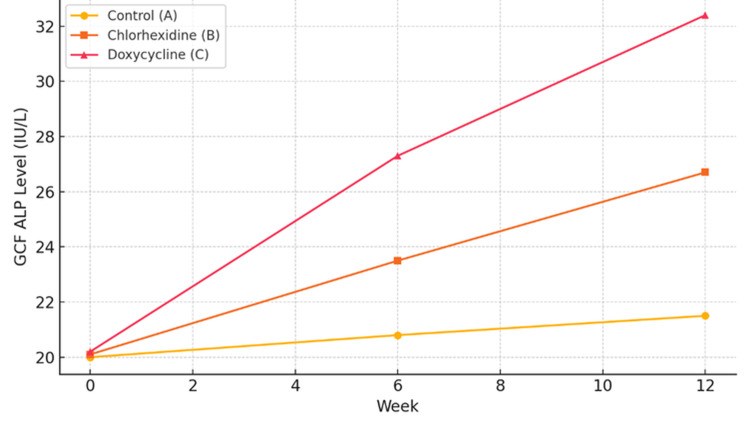
GCF ALP levels to estimate tooth movement GCF: gingival crevicular fluid, ALP: alkaline phosphatase

**Figure 2:**
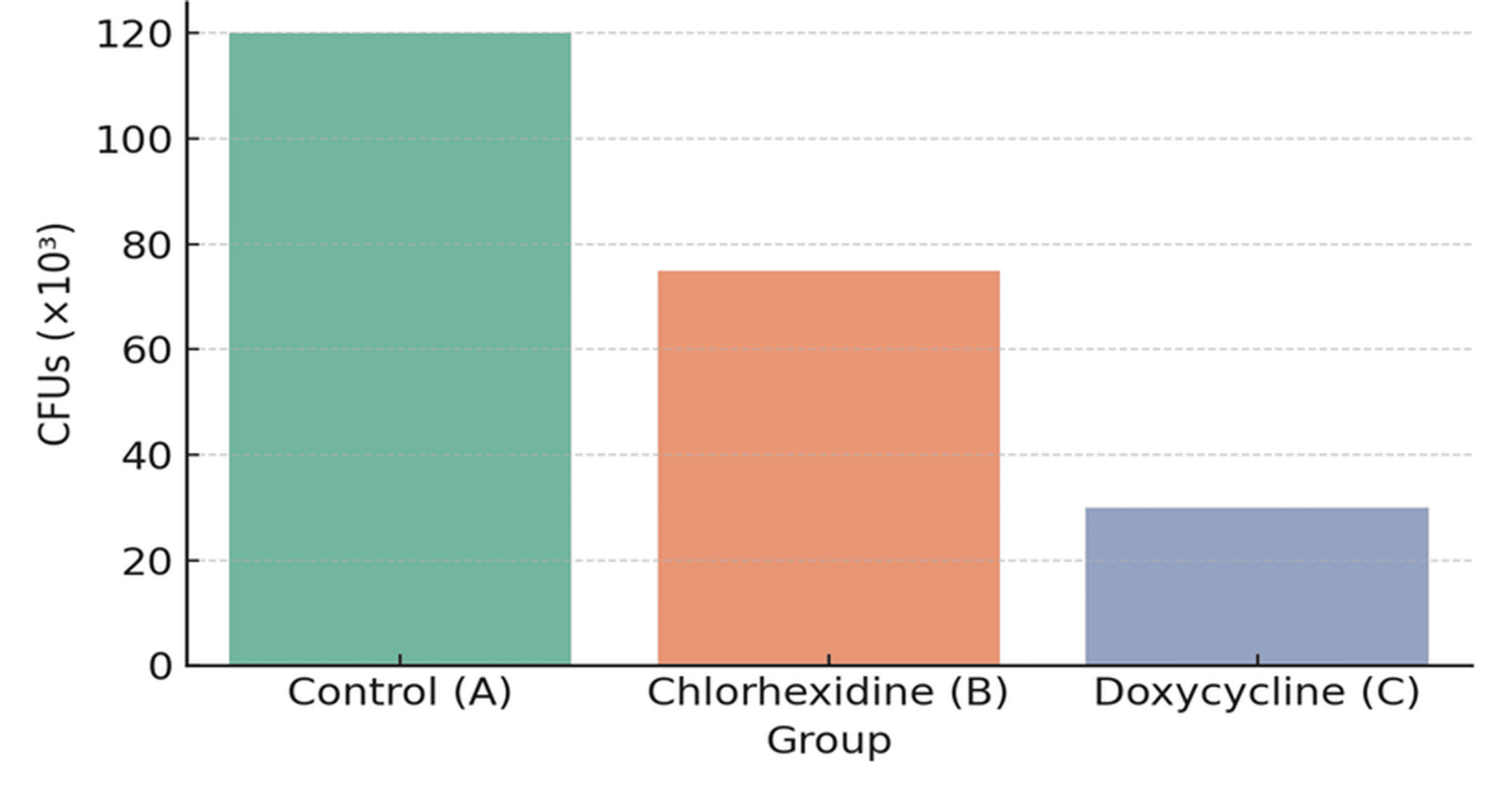
CFUs on aligner surface CFUs: colony-forming units

Pearson correlation analysis revealed a strong positive correlation between PI and GI (r = 0.912, p < 0.0001), as well as strong negative correlations between ALP and both CFUs (r = -0.723, p < 0.001) and GI (r = -0.807, p < 0.0001). Aligner transparency loss was inversely correlated with ALP levels (r = -0.788, p < 0.0001), indicating that a better biological response was associated with reduced microbial contamination and clearer aligners. These relationships are summarized in Table 5 and illustrated in Figure 1 (GCF ALP trends) and Figure 2 (CFU colonization patterns).

**Table 5:** Pearson correlation coefficients between clinical, microbial, and biochemical parameters PI: plaque index, GI: gingival index, GCF: gingival crevicular fluid, ALP: alkaline phosphatase

Correlated variables	Pearson's r	p-value	Strength of correlation	Direction
PI vs. GI	0.912	<0.0001	Very strong	Positive
GI vs. GCF ALP level	-0.807	<0.0001	Strong	Negative
CFU count vs. GCF ALP level	-0.723	<0.001	Moderate to strong	Negative
Transparency loss vs. GCF ALP level	-0.788	<0.0001	Strong	Negative

## Discussion

The findings from this study underscore the comprehensive clinical relevance of SADD as a disinfectant for clear aligners (Table 1 and Figure 1). Group C (SADD) showed a statistically significant reduction in PI and GI compared to groups A (control) and B (CHX), reinforcing its potent anti-inflammatory and antimicrobial effects. These outcomes are consistent with the mechanism of SADD involving inhibition of MMPs, which protect periodontal tissues from degradation [5].

In terms of microbial control, Table 2 and Figure 2 reveal that CFU counts were significantly lower in the SADD group than in the other groups. This aligns with prior research demonstrating doxycycline’s efficacy against a wide spectrum of oral pathogens, including *Streptococcus mutans* and *Candida albicans* [6,7]. Unlike traditional rinses that often fail to penetrate mature biofilms, doxycycline appears to suppress biofilm formation while maintaining biocompatibility.

Aligner aesthetics are critical in clear aligner therapy. According to Table 3, the doxycycline group experienced the least discoloration, with only 10% of samples exhibiting moderate-to-severe staining. This contrasts sharply with CHX, known for its staining potential [6]. These results position doxycycline as a superior esthetic-preserving agent.

Biochemically, the elevated GCF ALP levels in group C (Table 4) indicate enhanced osteoblastic activity, suggesting a possible acceleration in orthodontic tooth movement. This finding supports the theory that SADD may aid in modulating bone turnover while maintaining periodontal stability [5,8].

Correlation analysis presented in Table 5 further validates the interrelationship among microbial load, inflammation, and bone biology. Notably, ALP levels showed strong negative correlations with CFU counts, PI, GI, and aligner staining, reinforcing that effective microbial control may positively influence both esthetic and biological outcomes.

Strengths and limitations

This study’s strengths include its well-controlled design, integration of clinical, microbiological, biochemical, and esthetic endpoints, and use of real-world interventions. However, limitations exist. The sample size was modest, and the 12-week duration may not capture long-term trends. The visual transparency assessment could have been complemented with advanced imaging, and patient-reported outcomes, such as taste, comfort, and adherence, were not recorded.

Thus, sub-antimicrobial dose doxycycline demonstrated superior performance compared to chlorhexidine, providing dual benefits of microbial suppression and biological activation while preserving aligner esthetics. These findings support the integration of SADD in aligner hygiene protocols. Future studies should include larger cohorts, extended follow-ups, and molecular-level analysis to validate and expand upon these promising outcomes.

## Conclusions

The findings of this study demonstrate that SADD, when employed as a daily aligner soak, offers distinct advantages over traditional antimicrobial agents. Doxycycline not only significantly reduces microbial biofilm and plaque-induced gingival inflammation but also enhances biological responses related to bone remodeling, as reflected by elevated GCF ALP levels. Furthermore, the minimal staining of aligners observed in the doxycycline group suggests improved long-term esthetic preservation, which is crucial for patient compliance.

Clinically, these results support the potential use of SADD as an adjunctive pharmacological agent in aligner-based orthodontics, simultaneously promoting periodontal health, controlling biofilm, and possibly enhancing the biological efficiency of tooth movement. Given the rising demand for minimally invasive, esthetic, and biologically compatible treatment solutions, doxycycline-based aligner disinfection protocols may represent a promising addition to contemporary orthodontic practice.
